# First Latin uterine transplantation: we can do it!

**DOI:** 10.6061/clinics/2016(11)01

**Published:** 2016-11

**Authors:** José Maria Soares, Dani Ejzenberg, Wellington Andraus, Luiz Augusto Carneiro D'Albuquerque, Edmund Chada Baracat

**Affiliations:** I Hospital das Clínicas da Faculdade de Medicina da Universidade de São Paulo, Departamento de Obstetrícia e Ginecologia, Disciplina de Ginecologia, São Paulo/SP, Brazil; II Hospital das Clínicas da Faculdade de Medicina da Universidade de São Paulo, Departamento de Gastroenterologia, Disciplina de Transplantes de Órgãos do Aparelho Digestivo, São Paulo/SP, Brazil

Reproductive techniques are improving the rate of successful pregnancy. However, there is great concern regarding the uterine factor, particularly after hysterectomy or in the case of congenital absence of this organ. In fact, these issues may affect between 3 and 5% of the general population 1, and regrettably, there is no treatment for this type of condition. However, uterine transplantation may provide hope for women with this source of infertility 2.

Adoptions or surrogacy are two options for women who lack a uterus. However, both of these alternatives may be difficult for women to accept: a) in the first case, most women may want to produce their own, genetically related child, and b) in the second case, women need to find a relative or other individual willing to undergo pregnancy. Additionally, in the latter case, emotional ties could develop in relation to the baby. This situation is of great concern and limits women’s willingness to choose this option 2. In addition, there is a juridical problem in certain countries, such as Japan and Sweden: that is, the law prohibits surrogacy 2. Therefore, the only option left for these women is the possibility of a uterine transplant.

Over the last seven decades, several animal experimental models have been used, such as mice 3, rats 4, rabbits 5, sheep 6, pigs 7 and primates 8. Successful pregnancies were not reported in early studies due to the difficulty of the techniques used and immunological problems related to allotransplantation. These problems were also initially the main limitations for human uterine transplantation 2. Later, however, the evolution of immunosuppressants and microsurgical techniques increased the expectation of success and resulted the first report of a related birth in Sweden in 2014 9. Subsequently, three more births were reported, and others may have also resulted from the series of published cases 9-11. However, the record of uterine transplantation is imperfect, with unsuccessful pregnancies being reported in certain cases. For example, in a trial in Turkey, certain women attained positive pregnancy results but no live births 12.

Cadaver organ donation represents a source for many women. However, there are often impediments to use of these organs in transplantation: costs, religious concerns, tissue viability, preservation of the organ to be transplanted, trained professionals (a long learning curve is required) and family consent. The last consideration is a great barrier in many countries 13-14. The first relevant report worldwide involved a 22-year-old Turkish woman who received a uterus from a dead donor in 2011 12. The second report described a transplantation performed at the Cleveland Clinic in 2016; this was the first American surgical trial of uterine transplantation in a woman of reproductive age 15. Recently, in September 2016, a Brazilian team also attempted cadaver donor transplantation in this context. The surgery was specifically conducted by the Hepatic Transplantation Group in association with the Gynecology Division at Hospital das Clínicas da Faculdade de Medicina de São Paulo. It was a considerable challenge, but it was done!

Regardless of the time required to include uterine transplantation in clinical routine, cadaver donor uterus transplantation may provide hope for women without a uterus. We can finally perform this procedure, and we have many positive expectations regarding this new frontier in human reproduction.
